# Impact of Peels Extracts from an Italian Ancient Tomato Variety Grown under Drought Stress Conditions on Vascular Related Dysfunction

**DOI:** 10.3390/molecules26144289

**Published:** 2021-07-15

**Authors:** Maria Michela Cesare, Francesca Felice, Veronica Conti, Luca Cerri, Ylenia Zambito, Marco Romi, Giampiero Cai, Claudio Cantini, Rossella Di Stefano

**Affiliations:** 1 Cardiovascular Research Laboratory, Department of Surgical, Medical and Molecular Pathology and Critical Care Medicine, University of Pisa, 56100 Pisa, Italy; maria.cesare@student.unisi.it (M.M.C.); rossella.distefano@unipi.it (R.D.S.); 2 Department of Life Sciences, University of Siena, Via P.A. Mattioli 4, 53100 Siena, Italy; conti30@student.unisi.it (V.C.); l.cerri3@student.unisi.it (L.C.); marco.romi@unisi.it (M.R.); giampiero.cai@unisi.it (G.C.); 3 Department of Pharmacy, University of Pisa, 56126 Pisa, Italy; ylenia.zambito@unipi.it; 4 Interdepartmental Research Center, Nutraceuticals and Food for Health, University of Pisa, 56100 Pisa, Italy; 5 CNR-IBE (Consiglio Nazionale delle Ricerche-Istituto per la Bioeconomia), 58022 Follonica, Italy; claudio.cantini@ibe.cnr.it; 6 S.D. of Sport Medicine, Pisa University Hospital, 56100 Pisa, Italy

**Keywords:** tomato peel extract, tomato by-products, oxidative stress, drought stress, polyphenols, human vascular endothelial cells

## Abstract

Background: Tomato by-products contain a great variety of biologically active substances and represent a significant source of natural antioxidant supplements of the human diet. The aim of the work was to compare the antioxidant properties of a by-product from an ancient Tuscan tomato variety, Rosso di Pitigliano (RED), obtained by growing plants in normal conditions (-Ctr) or in drought stress conditions (-Ds) for their beneficial effects on vascular related dysfunction. Methods: The antioxidant activity and total polyphenol content (TPC) were measured. The identification of bioactive compounds of tomato peel was performed by HPLC. HUVEC were pre-treated with different TPC of RED-Ctr or RED-Ds, then stressed with H_2_O_2_. Cell viability, ROS production and CAT, SOD and GPx activities were evaluated. Permeation of antioxidant molecules contained in RED across excised rat intestine was also studied. Results: RED-Ds tomato peel extract possessed higher TPC than compared to RED-Ctr (361.32 ± 7.204 mg vs. 152.46 ± 1.568 mg GAE/100 g fresh weight). All extracts were non-cytotoxic. Two hour pre-treatment with 5 µg GAE/mL from RED-Ctr or RED-Ds showed protection from H_2_O_2_-induced oxidative stress and significantly reduced ROS production raising SOD and CAT activity (* *p* < 0.05 and ** *p* < 0.005 vs. H_2_O_2_, respectively). The permeation of antioxidant molecules contained in RED-Ctr or RED-Ds across excised rat intestine was high with non-significant difference between the two RED types (41.9 ± 9.6% vs. 26.6 ± 7.8%). Conclusions: RED-Ds tomato peel extract represents a good source of bioactive molecules, which protects HUVECs from oxidative stress at low concentration.

## 1. Introduction

The tomato (*Solanum lycopersicum* L.) is an edible Mediterranean plant already known for its beneficial properties. Whether it is fresh or processed, it is one of the most consumed vegetables worldwide [1,2] and a key component of the daily meals in many countries. Epidemiological studies have revealed a strong association between tomato consumption and decreased risk of chronic degenerative diseases such as cancer, cardiovascular and neurodegenerative pathologies [3,4,5,6,7]. In virtue of their high consumption, tomatoes and tomato products represent an important source of natural antioxidants, including carotenoids and phenolic compounds that are contained in the human diet [4,8,9,10].

Tomatoes are well known for their content in carotenoids such as lycopene as well as ascorbic acid, vitamin E and phenol compounds, in particular, flavonoids and hydroxycinnamic acids [11,12]. Lycopene and β-carotene are the main C40 carotenoids found in tomatoes [13], whilst the chemical class of polyphenols is represented by rutin (quercetin 3-*O*-rutinoside), quercetin, naringenin and chalconaringenin, which are just a few of the various flavonoids found in this fruit, together with organic acids such as benzoic, protocatechuic and cinnamic [14,15]. Many articles have reported the presence of flavonoids and hydroxycinnamic acids in tomatoes [16,17]. This group of polyphenols includes a variety of chemical structures that act on antioxidant enzymes such as superoxide dismutase (SOD), catalase (CAT) and glutathione peroxidase (GPx), resulting in very efficient peroxyl radicals scavenger activity [18]. The above are three key enzymes in the first line defense. They are very fast in neutralizing any potentially oxidative molecules in the cells [19].

Tomato plants adapt to different climatic conditions and to abiotic stress, such as the shortage of water which is one of the most serious problems concerning crops [20]. Unfortunately, tomato plants are highly sensitive to drought stress. The lack or shortage of water is the most common environmental factor that influences plant growth and productivity/yield [21]. In the future, water will become a strategic resource and, therefore, industrial and agricultural processes requiring low water consumption must be developed.

Plants have adopted various strategies to cope with water deficiency, including changes in growth and development [22,23]. In normal conditions, antioxidant defense systems are in a dynamic balance in plant cells, whereas stress conditions may result in an increase in antioxidant compounds that are either enzymatic or non-enzymatic in nature [24,25]. Water shortage stress can affect the yield of tomato crops, the volume, diameter and composition of the fruits, e.g., lycopene and total soluble sugars content [26].

Moreover, what is remarkable is the amount of various kinds of useful waste products, such as peels (about 60%) and seeds (around 40%). During the industrial processing of tomatoes, large quantities of by-products are generated, such as peels, which have high nutritional value [27]. Many studies have tested their potential beneficial effects on health [28,29,30] and optimized the extraction process of carotenoids from tomato skins [31]. Moreover, it is important to recycle industrial tomato processing waste to reduce the environmental impact of agro-industrial transformation processes.

Hydroxycinnamic acids, such as chlorogenic, caffeic, ferulic and p-coumaric, contribute to the antioxidant ability of tomatoes and their derivatives. HUVEC is an excellent model to study a broad array of diseases, including cardiovascular and metabolic diseases [32] so several studies have tested antioxidant molecules derived from natural products on HUVEC by in vitro experiments related to vascular dysfunction [28,33,34,35].

The aim of the present study was to compare the antioxidant properties of a by-product from an ancient Tuscan tomato variety, Rosso di Pitigliano, which is obtained by growing plants in normal conditions or in drought stress conditions. To this purpose an in vitro model of human endothelial vascular cells, the HUVEC, was used. The Rosso di Pitigliano variety was chosen from nine local Tuscan varieties in virtue of its highest concentration of antioxidants [36]. Furthermore, the extracts were evaluated both in vitro and ex vivo in order to compare their beneficial effects on vascular related dysfunction following oral intake.

## 2. Results

### 2.1. Characterization and Bioactive Compounds Profile of Tomato Peel Extracts

Antioxidant activity, which is measured by the ferric-reducing antioxidant power (FRAP) assay, and TPC, which is measured by Folin-Ciocalteu assay, are reported in Table 1. Although reference is made to TPC, it should be kept in mind that the Folin-Ciocalteau reagent is not specific for phenolic compounds and could also react with other molecules present in the extract with reducing characteristics. The data revealed that plants grown in drought stress condition had a higher TPC than plants grown in normal conditions, while no significant difference was found between the two extracts in the antioxidant activity as determined with the FRAP assay.

The identification of bioactive compounds of tomato peel was performed by high performance liquid chromatography (HPLC) analysis. The content of lycopene, vitamin C, rutin, caffeic acid and naringenin is reported in Table 2.

No significant differences were observed between the peel of the Rosso di Pitigliano (RED) growth in normal condition (-Ctr) and that of the RED growth in drought stress condition (-Ds) for all compounds, except for lycopene. However, the contents in rutin, caffeic acid and naringenin were moderately increased in RED-Ds with respect to RED-Ctr peel extracts. Neither quercetin nor chlorogenic acid could be detected.

### 2.2. Dose and Time Dependent Response of Cell Viability to Tomato Peels Extracts

**Figure 1:**
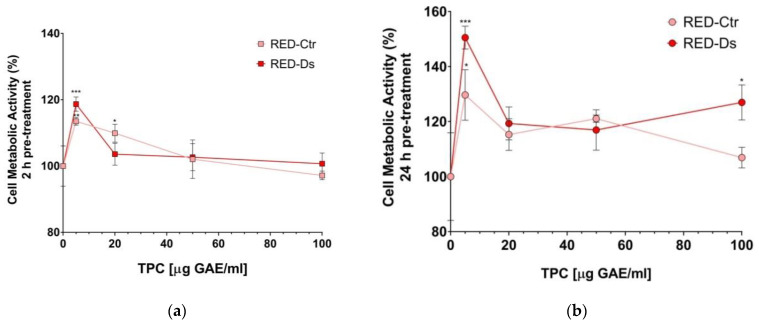
Dose-dependence and time-dependence of cell metabolic activity. HUVEC were cultured 2 h (**a**) or 24 h (**b**) with different concentrations of total polyphenols contained (TPC) in Rosso di Pitigliano (RED) tomato peel extracts (5-20-50-100 µg GAE/mL), which are obtained under normal plant growing conditions (Ctr) or in dehydration conditions (Ds). Data are expressed as % of metabolically active cells on an untreated cell basis (control). Graphical data are represented as mean ± SD of three separate experiments run in triplicate. * *p* < 0.05, ** *p* < 0.005, *** *p* < 0.0001 vs. Control (untreated cells).

Cell viability was evaluated by the WST-1 colorimetric assay. HUVEC was treated for 2 h or 24 h with different concentrations of TPC of RED-Ctr or RED-Ds tomato peel extracts (5-20-50-100 µg GAE/mL). The data obtained after 2 h or 24 h of treatment (Figure 1a,b) show the non-toxicity of extracts at all concentrations tested. Significant support to cell viability was observed after 24 h of treatment with 5 µg GAE/mL of RED-Ds extracts compared to the control (*p* < 0.0001), demonstrating that the presence of the extracts prevents the decay of cell viability which occurs after 24 h of culture.

### 2.3. Protective Effect against Oxidative Stress

**Figure 2:**
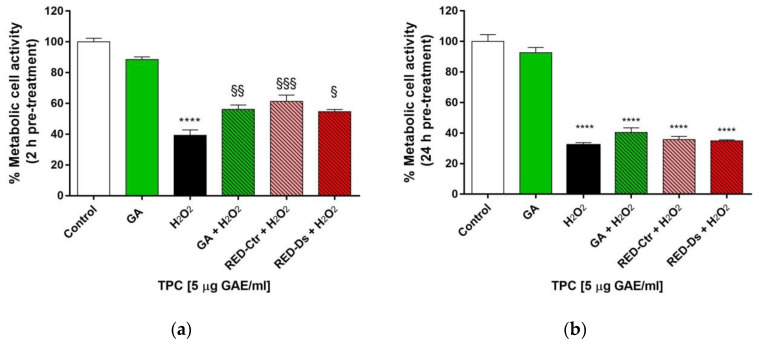
Cell viability after (**a**) 2 h or (**b**) 24 h pre-treatment with 5 µg GAE/mL TPC from Rosso di Pitigliano peel extracts or with 5 µg/mL gallic acid (GA) as positive control and the subsequent 1 h treatment with H_2_O_2_ (100 μM). RED-Ctr: plants grown in normal condition; RED-Ds: plants grown in drought stress condition. **** *p* < 0.0001 vs. Control (untreated cells); § *p* < 0.05, §§ *p* < 0.005 and §§§ *p* < 0.0005 vs. H_2_O_2_.

In order to evaluate the protective effect of tomato peel extracts against oxidative stress, we used the active antioxidants concentration of 5 µg/mL GAE. The same concentration of standard gallic acid (GA) was used as positive control. The data show that HUVEC treatment with H_2_O_2_ significantly reduced the viable cell number, expressed as % of metabolic cell activity on an untreated cells basis. The results showed a protective effect after 2 h pre-treatment with both RED-Ctr and RED-Ds tomato peel extracts (*p* < 0.0005 and *p* < 0.05 vs. H_2_O_2_ treated cells, respectively), but no protective effect after 24 h pre-treatment (Figure 2a,b). The same concentrations of standard GA protects the cell after 2 h (*p* < 0.005 vs. H_2_O_2_), but not after 24 h pre-treatment.

### 2.4. Reactive Oxygen Species Production

The antioxidant activity of the bioactive molecules in the tomato peel was assessed through the evaluation of ROS production, both with and without H_2_O_2_-stress induction on HUVEC.

**Figure 3:**
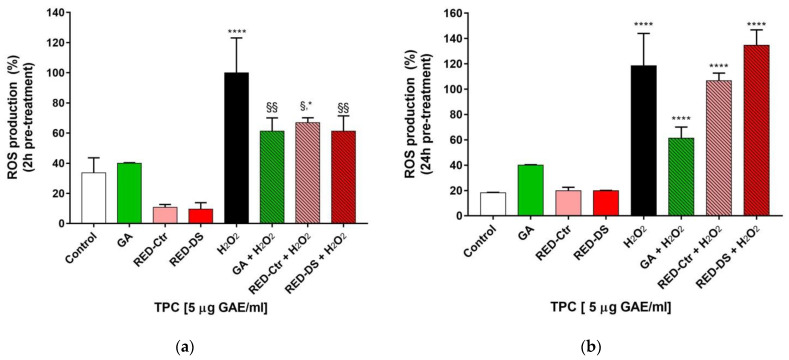
Reactive Oxygen Species (ROS) production in HUVEC pre-treated with 5 µg GAE/mL of total polyphenol content (TPC) of Rosso di Pitigliano peel extracts or with 5 µg/mL gallic acid (GA) as positive control for 2 h (**a**) and 24 h (**b**) and with H_2_O_2_ (100 µM) for 1 h. RED-Ctr: plants grown in normal condition; RED-Ds: plants grown in drought stress condition. Data are expressed as % ROS production and are representative of three separate experiments run in triplicate. * *p* < 0.05 and **** *p* <0.0001 vs. Control (untreated cells); § *p* < 0.05, §§ *p* < 0.005 vs. H_2_O_2_.

ROS accumulation in HUVEC was evaluated after 2 h or 24 h pre-treatment with 5 μg GAE/mL of tomato peel extracts. The same concentration of standard GA was used as the positive control. Treatment of HUVEC with H_2_O_2_ significantly increased intracellular ROS production compared with untreated cells (*p* < 0.0001). As shown in Figure 3a, after 2 h pre-incubation, RED-Ds reduced ROS production significantly than compared with H_2_O_2_ treatment (*p* < 0.005). On the contrary, the 24 h pre-treatment with either RED-Ctr or RED-Ds did not prevent the increase in ROS production induced by H_2_O_2_ (Figure 3b). On its part, 5 µg/mL GA exerted a protective effect only after 2 h pre-treatment.

### 2.5. Antioxidant Enzimes Activity

**Figure 4:**
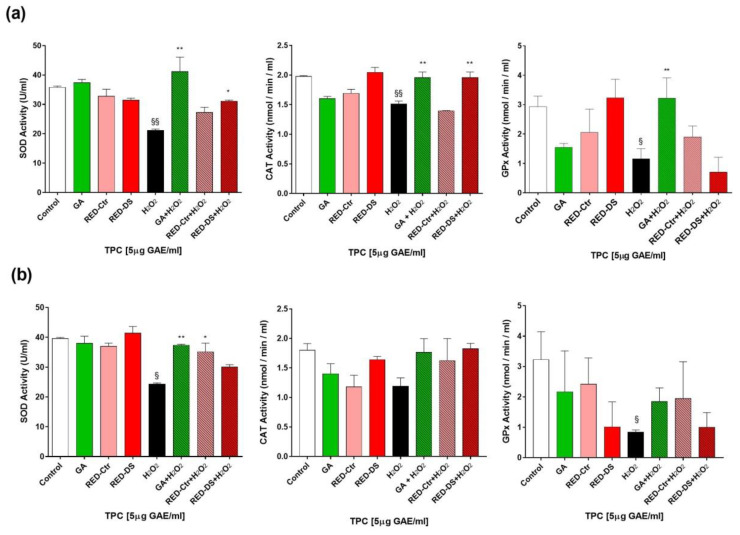
Activity of SOD, CAT and GPx enzymes in HUVEC pre-treated for 2 h (**a**) and 24 h (**b**) with 5 µg GAE/mL of tomato peel extracts or with 5 µg/mL gallic acid (GA) as positive control and with H_2_O_2_ (100 µM) for 1 h. RED-Ctr: plants grown in normal condition; RED-Ds: plants grown in drought stress condition. * *p* < 0.05, ** *p* < 0.005 vs. H_2_O_2_; § *p* < 0.05, §§ *p* < 0.005 vs. Control.

The antioxidant activity was evaluated by measuring SOD, CAT and GPx enzymes activity in HUVEC pre-treated for 2 h (Figure 4a) or 24 h (Figure 4b) with 5 µg GAE/mL of tomato peel extracts or with 5 µg/mL GA as positive control and with H_2_O_2_ (100 µM) for 1 h. In Figure 4a, treatment of HUVEC with H_2_O_2_ appears to significantly reduce the antioxidant enzymes activity compared with untreated cells (*p* < 0.005). Nevertheless, 2 h pre-treatment of cells with RED-Ds prevented the negative effect of H_2_O_2_ by acting on SOD and CAT enzymes, which are known to be involved in the first line defense system against ROS. No effect of RED-Ds is observed in Figure 4b on CAT and GPx activity after 24 h pre-treatment, whereas RED-Ctr was observed to act on SOD activity. The GA positive control showed a protective effect after either 2 h or 24 h pre-treatment by acting on CAT, SOD and GPx after 2 h and on SOD after 24 h pre-treatment.

### 2.6. Studies on the Permeation of Antioxidant Molecules Contained in RED across Excised Rat Intestine

The excised jejunal rat epithelium was chosen among the ex vivo intestinal models for the studies of antioxidant molecules permeability because its tight junctions are similar in tightness and number to those of the human jejunum [37].

**Figure 5:**
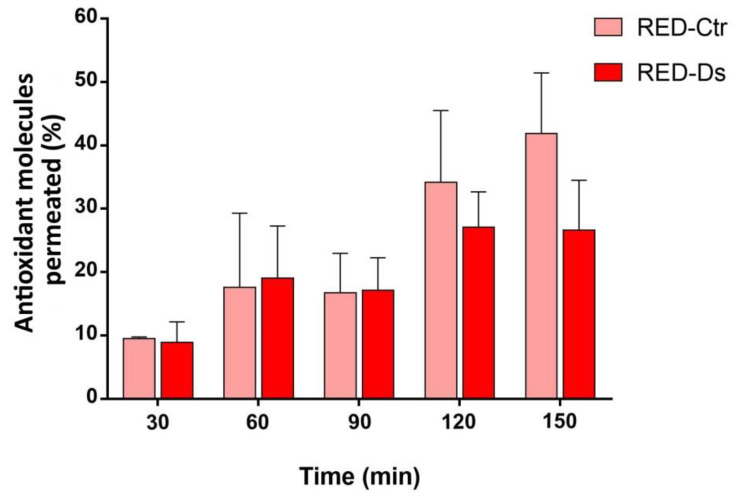
Data on the permeation of antioxidant molecules contained in RED-Ctr or RED-Ds in phosphate buffer, pH 7.4, 0.13 M, at the equivalent gallic acid concentration (GAE) of 110 µg/mL across the excised jejunal rat epithelium (*n* = 4).

The data on the permeation of antioxidant molecules contained in either RED-Ctr or RED-Ds across the excised jejunal rat epithelium, reported in Figure 5, show a non-significant difference in antioxidant molecules fraction permeated between the two extracts being compared. However, it appears that from comparing the mean RED-Ctr and RED-Ds cumulative permeated fractions (41.9 ± 9.6% vs. 26.6 ± 7.8%), the antioxidants from RED-Ctr are more permeable. It is observed in Figure 5 that with RED-Ctr the antioxidant molecules fraction permeated gradually increases over time up to 150 min, whereas with RED-Ds a maximum in permeation is reached after 120 min. These trends might not be ascribed to the difference in the antioxidant molecule’s ability to permeate but, rather, to a difference in the molecules contained in the extracts. Indeed, the Clioxicarb that bubbled in the two hemi-cells during the permeation experiment made the environment strongly oxidizing and this could cause degradation of the antioxidant molecule as we had already observed with grape seed extracts [38]. A more marked oxidation of the antioxidant contained in RED-Ds than those contained in RED-Ctr was observed, which implies a higher antioxidant potential of the former extract. At any rate, the data in Figure 5 show an ability of the antioxidants contained in the extracts under study to permeate across the intestinal epithelium and also in the free form, while not encapsulated in release systems that are able to protect them from degradation [34]. By the method of the stability study, contrary to what observed with cherry extracts, it had been observed that at the end of the experiment, i.e., after 4 h, no degradation of the antioxidant molecules contained in either RED-Ctr or RED-Ds was observed (data not shown). However, it should be considered that, unlike the ex vivo experiments, no Clioxicarb was used in this experiment.

## 3. Discussion

Tomato is an important component of the Mediterranean diet. Its cultivation is largely focused in semi-arid Mediterranean zones, where it needs to be cultivated under irrigation and where drought events related to climate change are generally more frequent [39].

Moreover, the management of by-products obtained during tomato processing is one of the most important issues related to environmental sustainability. If these by-products remain unused, there is a problem with their disposal and also it is an expensive process for companies. One way to avoid this problem is to reuse tomato processing by-products. Tomato by-products contain a great variety of biologically active substances, which have been demonstrated by in vitro and in vivo studies to possess antioxidant, hypolipidemic and anticarcinogenic properties [8]. Most of the functional compounds are derived from the secondary metabolism of plants and are synthesized in response to biotic stresses, as well as during normal physiological processes. Drought stress is one of the most impacting factors that seriously alters the plant physiology [40]. One of the first relevant reactions observed under drought is the enhancement of the antioxidant apparatus of the plant [41]. Some works have described an increase in biomolecules as well as in both antioxidant activity and total polyphenols in tomato plants subjected to water stress [42,43].

The present investigation is the first study where the antioxidant effects of the molecules contained in the peels of a variety of Tuscan tomatoes grown under normal conditions are compared with those obtained from peels of the same tomato variety grown under drought stress conditions.

In line with other studies [40,41,42,44,45], we observed the presence of a significantly higher content in bioactive compounds in the peel extracts of plants grown under water stress conditions compared to plants grown in normal conditions (361.32 mg GAE/100 g FW vs. 152.46 mg GAE/100 g FW). Moreover, a slight increase in vitamin C, rutin, caffeic acid and naringenin was observed in stressed plants. Our results suggest that water stress can affect peel TPC. The increase in TPC in RED-Ds peel extracts may suggest that plants are counteracting stress by synthesizing antioxidant molecules [44].

Lycopene is the main antioxidant in tomatoes for it is responsible for the red color [46] and represents more than 80% of total tomato carotenoid content in the fully ripened fruit. Lycopene is the major compound present in RED tomato peel. This bioactive compound has notable biological properties related to its antioxidant activity [47,48]. However, our data indicate that lycopene does not increase in plants subjected to drought stress but rather decreases. This is confirmed in other papers where drought stress was found to be capable of lowering the lycopene content compared to well irrigated plants [49,50]. Indeed, irrigation seems to have a pronounced effect on the biosynthesis of lycopene [51] and the differences may be due to the contrast in the responsive capacity of the genotypes to the different hydric conditions. In general, we agree that the various tomato crops respond differently and therefore generates a different concentration of metabolites when subjected to abiotic or biotic stress.

Several foods and dietary supplements have been shown to exert a protection against cardiovascular disease (CVD) as well as to the disorders related to oxidative stress, including allergy, cancer, diabetes, immune, inflammatory obesity and Parkinson’s diseases [52]. In this study, we have evaluated the protective effect of Tuscan tomato peel extracts on endothelial cells. Our results show that lower concentrations of RED significantly prevents the decay of cell viability after 24 h of treatment compared to untreated cells. Oxidative stress induced cell apoptosis when the endogenous antioxidant factors were decreased [53]. In particular, vascular oxidative stress contributes to the mechanisms of vascular dysfunction and has been stated to play an important role in a number of cardiovascular pathologies. In our study, we observed that low concentrations of TPC and the short-time of treatment of RED-Ds were sufficient to significantly reduce the effect of oxidative stress induced by hydrogen peroxide and to produce cytoprotective effects in HUVEC, as well as a significant reduction in ROS production, when compared with untreated cells. Our results demonstrate a direct antioxidant potential of RED-Ds peel extract and this is in agreement with the well described general quenching activity of carotenoids against hydrogen peroxide [54]. Moreover, our data show that the antioxidant properties of RED-Ds are due to its effect on SOD and CAT enzyme activity.

In this work the extract obtained under water-shortage stress conditions, RED-Ds, has shown to possess antioxidant activity on HUVEC, i.e., on the cells of the endothelium of blood vessels and thus could be potential candidate compounds for the prevention of CVD.

Furthermore, the protective effects are mainly due to the presence of bioactive compounds such as carotenoids, vitamins and polyphenols, which probably interacts synergistically [55]. Various studies have demonstrated that both concentrations and combinations of antioxidants can influence the synergistic antioxidant ability. Indeed, the biological activity of lycopene can be enhanced by the presence of other active antioxidant compounds such as vitamin C, β-carotene or vitamin E [56,57,58]. Nevertheless, particular concentrations and combinations of antioxidants can also exert antagonistic effects [59]. Thus, the cellular protective effect of the RED-Ds peel extracts may be due to proper concentrations and combinations of antioxidants found in the Ds peels. Furthermore, since the antioxidant molecules permeation was followed in the permeation experiments, we can hypothesize that these bioactive molecules can reach the target site in sufficient concentration to carry out their protective action on endothelial cells. In fact, in the in vitro model both in the RED-Ctr and RED-Ds cases, a permeation of the total polyphenols is observed at about 30% of the applied dose (110 µg GAE/mL) and the experiments on HUVEC showed that the polyphenols are already active at a concentration of 5 µg GAE/mL.

Natural extracts have shown their effects against chronic diseases, including CVD. For the treatment of CVD, prevention plays an important role and the introduction of nutraceuticals into the diet could represent the first defense mechanism of the body from oxidative stress. Nevertheless, the current coronavirus (COVID-19) pandemic has highlighted the importance of an appropriate nutrition with functional compounds that can offer further antiviral approaches to public health, optimizing the ability of the immune system to prevent and control pathogenic viral infections, as highlighted in recent works [60].

## 4. Materials and Methods

### 4.1. Materials

Hexane, acetone and Folin-Ciocalteau reagent were purchased from Sigma-Aldrich (Milan, Italy). H_2_O_2_ was bought from Farmac-Zabban S.p.a. (Calderara di Reno, BO, Italy); gelatin was obtained from Sigma-Aldrich (Milan, Italy). Medium 199 (M199), fetal bovine serum (FBS), penicillin-streptomycin solution, l-glutamine and HEPES buffer were supplied by Hospira S.r.l. (Naples, Italy). 4-[3-(4-iodophenyl)-2-(4-nitrophenyl)-2H-5-tetrazolium]-1,3-benzenedisulfonate (WST-1 assay) was purchased from Roche Applied Science (Mannheim, Germany); 5-(and-6)-chloromethyl-2′,7′-dichloro-di-hydro-fluorescein diacetate, acetyl ester (CM-H_2_DCFDA) was supplied by Invitrogen (Thermo Fisher Scientific, Vantaa, Finland).

### 4.2. Sample Preparation

#### 4.2.1. Fruit Harvesting and Stress Condition

The tomato variety used for the study was chosen from nine local Tuscan varieties (*Solanum lycopersicum* L.) registered at the Tuscan Regional Germplasm Bank and characterized by different morphological and agronomic features. The tomato variety used for this study was Rosso di Pitigliano obtained by growing plants in normal conditions or in drought stress condition. Four plants were used for the control and four for the stress condition, which are cultivated in a greenhouse (Botanical garden, University of Siena, Siena, Italy) and all plants were well-watered until the beginning of the water stress treatment. The drought stress conditions began when the first fruits started to grow. The plants were around 120 cm high at the beginning of the stress test and the waterless treatment lasted for 20 days [61,62]. Ripe tomatoes (*Solanum lycopersicum* L.) were harvested in the period between 1 August to 31 August 2019 and fruits were refrigerated at −80 °C to stop their normal biological processes.

#### 4.2.2. Tomato Peel Extracts Preparation

Lyophilized tomato peel sample was prepared according to Beconcini et al. [34], with some modifications. Briefly, frozen tomatoes were hand peeled and weighed to obtain 2 g of tomato peel and then 6 mL of water was added. The mixture was homogenized (Ultra-Turrax^®^ T25 based IKA, Saint Louis, MS, USA) for about 3 min and sonicated (Elma Transsonic T 460/H, Wetzikon, Switzerland) for 20 min to guarantee complete cellular decomposition. The mix was again homogenized one minute and centrifuged 5 min at 13,000 rpm (Eppendorf^®^ 5415D centrifuge, Hamburg, Germany) to separate the biomolecules from the pellet. The supernatant was filtered through a 0.45 µm cellulose acetate membrane filter (Sartorius, Göttingen, Germany). Finally, the tomato peel extracts were freeze-dried 48 h (freeze dryer LIO 5P, 5pascal, Italy). The freeze-dried tomato peel extracts were transferred into airtight containers and stored at −20 °C. The freeze-dried tomato peel extracts were diluted in water for further analysis.

### 4.3. HPLC Characterization

#### 4.3.1. Antioxidant Molecules

Rutin, quercetin, naringenin and caffeic acid were determined by HPLC (Perkin Elmer Nelson 3200 Series) with an RP-C18 column (SUPELCO Kromasil 100A-5u-C18 4.6 mm × 250 mm). Extraction was conducted in agreement with Tokusoglu et al., 2003 [63], although with some differences. One gram of each fruit peel was added to 1 mL of 70% acetone containing 1% (*v*/*v*) of HCl and 0.02 mg/mL of TBHQ (tert-Butylhydroquinone). Then, the mix was homogenized by Ultra-Turrax (IKA^®^) and 0.2 mL of HCl (1.2 M) was added. The mixture was incubated 2 h at 90 °C under continuous stirring. The sample was then cooled to room temperature and sonicated for 3 min. Finally, the extract was centrifuged 5 min at 3000× *g* and filtered through a 0.45 µm membrane filter. The HPLC measurements were performed in agreement with Kumar et al., 2008 [64], with slight differences. The mobile phase was composed of 2 solvents: water (A) and acetonitrile with 0.02% trifluoracetic acid (TFA) (B), with a linear gradient elution of 80% A and 20% B (0–5 min), 60% A and 40% B (5–8 min), 50% A and 50% B (8–12 min), 60% A and 40% B (12–17 min) and 80% A and 20% B (17–21 min). The flow rate was 1 mL/min and the absorbance was set at 365 nm for rutin and quercetin, 325 nm for caffeic acid and 280 nm for naringenin in a run time of 21 min. For the quantification of rutin, quercetin, naringenin and caffeic acid, respective standard calibration curves were used with each consisting five points obtained from standards in the 5–80 µg/mL concentration range (Sigma Chemical, St. Louis, MI, USA).

#### 4.3.2. Lycopene

The extraction of lycopene was conducted according to Barba et al., 2006 [65]; 0.3 g of tomato peel was added to 10 mL of hexane/acetone/ethanol (50:25:25 *v*/*v*/*v*) and the mix homogenized with Ultra-Turrax (IKA^®^). Then, 1.5 mL of distilled water was added followed by vortexing. One ml of the upper layer was dried under vacuum and the dry extract was resuspended in 0.4 mL of solution tetrahydrofuran (THF)/acetonitrile (ACN)/methanol (15:30:55 *v*/*v*/*v*). The mobile phase for HPLC consisted of methanol/ACN (90:10 *v*/*v*) and triethanolamine (TEA) 9 mM at a flow rate of 0.9 mL/min in a RP-C18 column (SUPELCO Kromasil 100A-5u-C18 4.6 mm × 250 mm) at an absorbance of 475 nm and in a run time of 20 min. For quantification, a standard calibration curve consisting of five points at the increasing concentrations of 6.25, 12.5, 25, 50 and 100 µg/mL of lycopene standard (Sigma Chemical, St. Louis, MI, USA) was used.

#### 4.3.3. Vitamin C

Ascorbic acid was extracted from 1 g of tomato peel dispersed in 2 mL of distilled water followed by homogenization with Ultra-Turrax (IKA^®^) and filtration through a 0.45 µm membrane filter [66]. For the HPLC method, an RP-C18 column (SUPELCO Kromasil 100A-5u-C18 4.6 mm × 250 mm) was used. The mobile phase consisted of 0.01 mol/L KH_2_PO_4_ buffer solution (pH = 2.6 with o-phosphoric acid), with a flow rate of 0.5 mL/min and a detection absorbance set at 250 nm. For quantification, a standard calibration curve consisting of five points at the increasing concentrations of 6.25, 12.5, 25, 50 and 100 µg/mL of ascorbic acid standard (Sigma Chemical, St. Louis, MI, USA) was used.

### 4.4. Antioxidant Activity

The total antioxidant potential of tomato peel extracts was determined using the FRAP assay reported by Benzie and Strain [67]. This is based on the reduction in Fe^3+^-2,4,6-Tri(2-pyridyl)-s-triazine (TPTZ) to a blue-colored Fe^2+^-TPTZ. Briefly, FRAP reagent was freshly prepared and the solution was heated at 37 °C for one hour. The absorbance was read at 593 nm using an UV-Vis spectrophotometer (Perkin Elmer, Lamba 25 spectrophotometer, Waltham, MA, USA). The FRAP values of the samples, which were expressed as µmol of Fe^2+^ per g of fresh weight (FW), were determined from a standards curve built up using ferrous sulphate.

### 4.5. Total Polyphenolic Content

The TPC in tomato peel extracts was determined in fresh fruit by the spectrophotometric method of Folin-Ciocalteau [68]. The absorbance was read at 765 nm. The results were obtained from a standard curve built up using gallic acid (GA) (Sigma-Aldrich) and were expressed as mg gallic acid equivalent (GAE) per 100 g FW.

### 4.6. HUVEC Isolation and Culture

HUVEC was harvested and isolated by enzymatic digestion as described by Jaffe et al. [69]. Human cells were obtained from discarded umbilical cords and treated anonymously; as such, approval from the University Ethics Review Board was not necessary. Isolated cells were centrifuged and the cell pellet was plated on gelatin pre-coated flasks and incubated for 24 h at 37 °C, 5% CO_2_ in Medium 199 (Life Sciences, Grand Island, NY, USA), which contained 10% fetal bovine serum (FBS), antibiotics (penicillin-streptomycin), growth factors (heparin, 50 U/mL and endothelial cell growth factor, 10 mg/mL) (all from Sigma-Aldrich, St. Louis, MO, USA), l-glutamine and HEPES buffer. After 24 h, the growth medium was replaced to remove the excess red blood cells.

### 4.7. Cell Treatment

The HUVEC between passage P2–P4 was treated as previously described [28]. Briefly, cells were treated for 2 h and 24 h with different concentrations of TPC (5, 20, 50 or 100 μg GAE/mL) of RED tomato peel extracts obtained from plants grown under standard conditions or under drought stress conditions, in growth medium with 5% FBS. Cells without treatment were used as a positive control. Then, the cells were washed with PBS and treated with 100 μM H_2_O_2_ for 1 h to induce oxidative stress. At the end of each treatment, the cells were analysed for viability and ROS production.

### 4.8. Cell Viability

After treating the HUVEC with the test samples or with H_2_O_2_ (100 μM), cell viability was assessed by WST-1 colorimetric assay, as previously described [28]. Briefly, at the end of the treatment, the cells were incubated with tetrazolium salt (10 μL/well) for 3 h at 37 °C in 5% CO_2_. Formazan dye formation was quantified by measuring the absorbance at 450 nm, with a multiplatform reader (Thermo Scientific Multiskan FC photometer for microplates, Thermo Fisher Scientific Oy, Vantaa, Finland). Absorbance was directly related to the number of metabolically active cells and viability was expressed as a percentage of viable cells.

### 4.9. ROS Production

ROS fluorescent probe CM-H_2_DCFDA, which is a cell permeable indicator for these compounds, was used to evaluate the intracellular production of ROS. As previously described [28], during the last 15 min of treatment with RED-Ctr, RED-Ds or H_2_O_2_, the HUVEC and CM-H2DCFDA (10 μM/well) dissolved in PBS and were co-incubated in the dark at room temperature. ROS production was detected by measuring the increase in fluorescence over time by the microplate reader (Fluorometer Thermo Scientific Fluoroskan Ascent Microplate). Fluorescence was measured by excitation at 488 nm and emission at 510 nm.

### 4.10. SOD, CAT and GPx Activities

After pre-treatment with 5 μg GAE/mL of RED-Ctr and RED-Ds for 2 h and 24 h with H_2_O_2_ (100 µM), the cells were scraped and lysated. The supernatant was collected and the CAT, SOD and GPx enzymatic activities were determined by commercially available kits, in accordance with the manufacturer’s instructions (Cayman Chemical Company, Ann Arbor, Michigan, MI, USA). The values were expressed in U/mL for SOD activity and nmol/min/mL for CAT and GPx activity.

### 4.11. RED Stability Studies

The stability of RED-Ctr and RED-Ds at pH 1.2 was assessed according to the procedure described in a previous study [34]. A solution of RED-Ctr or RED-Ds in simulated gastric fluid pH 1.2 (SGF) was equilibrated at 37 °C in a water bath under continuous stirring. At 30 min intervals over a total of 240 min, samples of 50 µL were withdrawn and analyzed for content in antioxidant molecules by the Folin-Ciocalteau method [68].

### 4.12. Studies on Permeation of Antioxidant Molecules Contained in RED across Excised Rat Intestine

These studies were carried out as described in previous papers [70,71]. Briefly, the intestinal mucosa was excised from non-fasted male Witstar rats weighing 250–300 g. All experiments were conducted under veterinary supervision and the protocols were approved by the scientific-ethical committee of the Italian University and Ministry of University and Research. The intestine was longitudinally cut into strips, rinsed free of luminal content and mounted in Ussing type cells (0.78 cm^2^ exposed surface) without removing the underlying muscle layer. After 20 min equilibration at 37 °C, RED-Ctr or RED-Ds in a concentration equivalent to 110 µg GAE /mL in phosphate buffer pH 7.4 (0.13 M) was added to the apical chamber. Three ml of fresh phosphate buffer pH 7.4 (0.13 M) was inserted in the acceptor compartment. Clioxicarb (95% O_2_ plus 5% CO_2_ mix) was bubbled in both compartments to ensure oxygenation of tissue and stirring. The apical to basolateral transport of tomato extracts was studied. At 30 min intervals of a total of 150 min, 100 μL samples were withdrawn from the acceptor and replaced with fresh pre-thermostated medium. The amount of antioxidant molecules permeated was determined by analyzing the withdrawn samples by the Folin-Ciocalteau method, as described previously [38]. The mean cumulative percentage permeated in a given time was calculated to plot each permeation profile.

### 4.13. Statistical Analysis

All results were presented as means ± standard deviation (SD) of at least three independent experiments. The difference among groups of values was evaluated by a one-way ANOVA and a post hoc analysis was performed by Turkey’s or Bonferroni’s multiple comparison test. In ex vivo experiments the significance of the difference between two values was evaluated by the Student’s *t*-test. Differences were considered significant, i.e., the null hypothesis was rejected for p values lower than 0.05. The GraphPad Prism Software v. 7.0 (GraphPad Software Inc., San Diego, CA, USA) was used for the statistical analysis of the data.

## 5. Conclusions

Mediterranean local varieties are a valuable inheritance to be re-evaluated, since they constitute an alternative source to breed for more resilient cultivars under extreme climate conditions.

In this study, tomato peel extracts of an ancient autochthonous Tuscan variety, which are obtained from plants grown in drought stress condition, showed enhanced antioxidant properties when cultured under water-shortage stress condition, which demonstrates potential industrially and pharmaceutically useful compounds for biomedical applications.

Since the tomato peel extracts have shown an antioxidant action on HUVECSs even at low concentrations and that a good percentage is able to permeate intact through the isolated rat intestine, it can be concluded that their supplementation in the diet may contribute to the prevention of cardiovascular diseases.

For this reason, the use of tomato peel extracts, especially those grown in drought stress conditions, can be an added value in nutraceuticals as their particular antioxidant composition can be useful for human health if included in the diet.

## Figures and Tables

**Table 1:** Rosso di Pitigliano (RED) tomato peel extract characterization.

Plant Growth Condition	Antioxidant Activity (µmol Fe^2+^/g Fresh Weight) ± SD	Total Polyphenols Content (mg GAE/100 g Fresh Weight) ± SD
Ctr	23.885 ± 0.375	152.46 ± 1.568
Ds	26.052 ± 0.556	361.32 ± 7.204 *

Plants were obtained under normal growth (Ctr) or under drought stress (Ds) conditions. * Significantly different from each other (*p* ˂ 0.05).

**Table 2:** Bioactive compounds profile of the tomato peel of RED varieties grown in different conditions.

Plant Growth Condition	Lycopene	Vitamin C	Rutin	Caffeic Acid	Naringenin
Ctr	171.0 ± 1.4 *	30.5 ± 8.81	11.60 ± 0.33	0.83 ± 0.08	1.13 ± 0.08
Ds	95.48 ± 6.39	39.6 ± 0	12.59 ± 0.14	1.19 ± 0.08	1.32 ± 0.03

Mean values and relative standard deviations are expressed as mg of compound per 100 g fresh weight. * Significantly different from one another (*p* ˂ 0.05). RED: Rosso di Pitigliano; Ctr: normal plant growth conditions; Ds: drought stress condition.

## Data Availability

Not applicable.

## Sample Availability

Samples of RED-Ctr and RED-Ds are available from the authors (M.M.C. and G.C.).
